# Understanding Pregnancy and Postpartum Health Using Ecological Momentary Assessment and Mobile Technology: Protocol for the Postpartum Mothers Mobile Study

**DOI:** 10.2196/13569

**Published:** 2019-06-26

**Authors:** Dara D Mendez, Sarah A Sanders, Hassan A Karimi, Pedram Gharani, Stephen L Rathbun, Tiffany L Gary-Webb, Meredith L Wallace, John J Gianakas, Lora E Burke, Esa M Davis

**Affiliations:** 1 Department of Epidemiology Graduate School of Public Health University of Pittsburgh Pittsburgh, PA United States; 2 Behavioral and Community Health Sciences Graduate School of Public Health University of Pittsburgh Pittsburgh, PA United States; 3 Geoinformatics Laboratory School of Computing and Information University of Pittsburgh Pittsburgh, PA United States; 4 Department of Epidemiology and Biostatistics College of Public Health University of Georgia Athens, GA United States; 5 Department of Psychiatry University of Pittsburgh Pittsburgh, PA United States; 6 Department of Health and Community Systems School of Nursing University of Pittsburgh Pittsburgh, PA United States; 7 Division of General Internal Medicine Department of Medicine University of Pittsburgh Pittsburgh, PA United States

**Keywords:** ecological momentary assessment (EMA), wireless technology, remote sensing technology, maternal health, pregnancy, postpartum, body weight, health status disparities, health equity

## Abstract

**Background:**

There are significant racial disparities in pregnancy and postpartum health outcomes, including postpartum weight retention and cardiometabolic risk. These racial disparities are a result of a complex interplay between contextual, environmental, behavioral, and psychosocial factors.

**Objective:**

This protocol provides a description of the development and infrastructure for the Postpartum Mothers Mobile Study (PMOMS), designed to better capture women’s daily experiences and exposures from late pregnancy through 1 year postpartum. The primary aims of PMOMS are to understand the contextual, psychosocial, and behavioral factors contributing to racial disparities in postpartum weight and cardiometabolic health, with a focus on the daily experiences of stress and racism, as well as contextual forms of stress (eg, neighborhood stress and structural racism).

**Methods:**

PMOMS is a longitudinal observation study that is ancillary to an existing randomized control trial, GDM^2^ (Comparison of Two Screening Strategies for Gestational Diabetes). PMOMS uses an efficient and cost-effective approach for recruitment by leveraging the infrastructure of GDM^2^, facilitating enrollment of participants while consolidating staff support from both studies. The primary data collection method is ecological momentary assessment (EMA) and through smart technology (ie, smartphones and scales). The development of the study includes: (1) the pilot phase and development of the smartphone app; (2) feedback and further development of the app including selection of key measures; and (3) implementation, recruitment, and retention.

**Results:**

PMOMS aims to recruit 350 participants during pregnancy, to be followed through the first year after delivery. Recruitment and data collection started in December 2017 and are expected to continue through September 2020. Initial results are expected in December 2020. As of early May 2019, PMOMS recruited a total of 305 participants. Key strengths and features of PMOMS have included data collection via smartphone technology to reduce the burden of multiple on-site visits, low attrition rate because of participation in an ongoing trial in which women are already motivated and enrolled, high EMA survey completion and the use of EMA as a unique data collection method to understand daily experiences, and shorter than expected timeframe for enrollment because of the infrastructure of the GDM^2^ trial.

**Conclusions:**

This protocol outlines the development of the PMOMS, one of the first published studies to use an ongoing EMA and mobile technology protocol during pregnancy and throughout 1 year postpartum to understand the health of childbearing populations and enduring racial disparities in postpartum weight and cardiometabolic health. Our findings will contribute to the improvement of data collection methods, particularly the role of EMA in capturing multiple exposures and knowledge in real time. Furthermore, the results of the study will inform future studies investigating weight and cardiometabolic health during pregnancy and the postpartum period, including how social determinants produce population disparities in these outcomes.

**International Registered Report Identifier (IRRID):**

DERR1-10.2196/13569

## Introduction

Research has consistently shown a racial disparity in postpartum weight retention, where black women are more likely to retain or gain weight after delivery compared with white women even when entering pregnancy at similar weights [1-4]. Multiple studies have attributed this disparity to individual-level factors, such as breastfeeding behavior [5,6], exposure to stressors [7], or diet and exercise [8,9], but these findings do not fully explain the racial disparity. Furthermore, there is a dearth of literature specifically addressing how contexts and environments intersect with individual-level factors in reproducing racial disparities. Given that stressful exposures to racism and related forms of oppression and discrimination are unique to black women and related to adverse perinatal outcomes [10], it is important to specifically understand how these stressors contribute to the disparity in postpartum weight retention and related cardiometabolic risks in the context of pregnancy.

The Postpartum Mobile Mothers Study (PMOMS) is an innovative longitudinal study designed to understand the contextual, behavioral, psychosocial, and clinical factors related to racial disparities in postpartum weight and cardiometabolic health. PMOMS includes pregnant populations recruited during midpregnancy and followed up through the first year postpartum and is ancillary to the Comparison of Two Screening Strategies for Gestational Diabetes (GDM^2^) trial [11]. PMOMS participants complete daily surveys via smartphone technology, weigh themselves via Bluetooth-enabled scales, and attend follow-up visits for anthropometric measurements. In this paper, we describe how PMOMS expands on the feasibility of using mobile technology in behavioral research via ecological momentary assessment (EMA) methods to understand women’s experiences and exposures in their natural environment via real-time measurements of psychosocial (eg, stress and racism), behavioral (eg, physical activity), and contextual (eg, location linked to neighborhood and environmental data) factors.

EMA is a well-known method in studying hypothesized environmental effects on human behavior and has been shown to be an effective method for regular or daily data collection [12]. EMA offers a way to understand experiences and exposures in real time, and often in the participants’ natural environments [13]. Mobile devices, such as smartphones, have become optimal vehicles for remote data collection or the collection of data in an environment that is not a controlled laboratory setting, including EMA data collection. When compared with data collection in a laboratory setting, remote, real-time data collection eliminates the need for long-term recall, considers the context in which people are responding, is consistent and reliable, has ecological validity, and provides opportunities for more data points [12,14]. In 1 study, physiological data collected via EMA to capture cardiovascular health, specifically blood pressure, produced different results from that collected in a laboratory context [15]. In addition, collecting EMA data has been shown to be feasible and accessible in various populations, with high participant satisfaction, and some studies showing completion rates of up to 89% [16-18].

There are several examples of EMA methods being used in clinical and public health research among childbearing or pregnant populations, the populations of focus for this study. Several studies included interventions focused on managing gestational weight gain and gestational diabetes [19-24]. For example, 2 studies [19,25] demonstrated how wireless glucometers contributed real-time blood glucose measurements, which helped to tailor a mobile app’s feedback to participants at risk for gestational diabetes. Observational studies, although fewer in number, have also demonstrated the feasibility and accuracy of using mobile technologies, including smartphones, to facilitate EMA data collection in a parous population [17,26,27].

One of the key EMA measures of interest in PMOMS includes reported experiences of racism, with a focus on interpersonal racism—including, microaggressions [28,29]. Several studies have used EMA approaches to specifically measure experiences of racism, discrimination, and other forms of marginalization [30-36], but none of these previous studies using EMA methods specifically addressed health during and after pregnancy. Furthermore, only one of these studies tracked EMA measurements over several months [31], whereas the others ranged between 3 days and 3 weeks [32-35]. Some studies used portable electronic devices to maintain data entries [31,36], whereas more recent studies incorporated smartphones [33-35] to understand various experiences of racism and discrimination. EMA data collected via mobile technology in this study allows us to query everyday experiences and momentary occurrences in a participant’s natural settings that may contribute to chronic exposure to racism.

Another key approach applied in this study is geographic momentary assessment (GMA), an extension of EMA, which measures location and environment in real time, providing an avenue to capture multiple environmental exposures over time. Geographic positioning systems (GPSs) are built into most modern smartphones, which allows for regular access to location information [37]. GMA methods allow researchers to match data collection points with the participant’s geographical location (ie, their natural environment) along with self-reported measures of contexts. The current GMA literature focuses mainly on behaviors such as substance use, where context, location, and environment may have a tremendous influence on outcomes. For example, some studies have assessed eating behaviors and substance use alongside measures of mental health and stress as mediators or predictors of these behaviors [37-40]. To date, no GMA studies have focused on childbearing populations, pregnancy, or the postpartum period.

This paper outlines the various processes and steps involved in designing and executing PMOMS, including how PMOMS is ancillary to an ongoing trial, GDM^2^; the use of mobile technology; applications of EMA methods; and longitudinal follow-up. Details of the research development process, infrastructure, challenges encountered, and lessons learned are also described.

## Methods

### Overview

We outline the GDM^2^ trial as the primary study in which PMOMS approaches and recruits potential participants; original research that contributed to the development of the present PMOMS methodology; the pilot studies conducted to confirm the feasibility of EMA methods among pregnant and postpartum women (ie, the PregEMA and PostpartumEMA pilot studies); and the technological infrastructure necessary for direct communication with participants and collection and transmission of participant data and related security measures.

### Comparison of the Two Screening Strategies for Gestational Diabetes (GDM ^2^) Trial: Parent Study

The key methodological and infrastructural element in PMOMS is its partnership with a parent study: the Comparison of Two Screening Strategies for Gestational Diabetes (GDM^2^) trial [11,41]. The GDM^2^ trial was designed to examine 2 testing strategies for screening and diagnosing gestational diabetes and to follow select women and infants through 12 months after delivery to assess metabolic risk profiles and infant growth. Given the similar study objectives and observation periods, PMOMS is an ancillary study to GDM^2^ and recruits directly from the parent study. GDM^2^ is as an excellent platform for PMOMS recruitment, including the interstudy collaboration, and is crucial to the success of PMOMS. Recruitment for GDM^2^ began in July 2015. Participants in the GDM^2^ trial are recruited and requested for consent during pregnancy, between 19 and 29 weeks’ gestation. After enrollment, participants are asked to attend 2 study visits during pregnancy to complete laboratory work, anthropometric measures, and brief questionnaires.

### Exemplar and Pilot Studies

The Advancing Real-time Data Collection with Adaptive Sampling and Innovative Technologies (EMPOWER) study served as an exemplary longitudinal EMA study for PMOMS as it was designed to understand factors related to relapse (of weight) among individuals enrolled in a weight loss intervention over 12 months [18]. The EMPOWER study incorporated EMA methods that provided guidance for PMOMS, but it also served as a precedent for the importance of longitudinal data collection regarding weight loss and retention. Although the PMOMS structure and population differ from those of the EMPOWER study, the findings helped to justify this long-term protocol [18]. The EMPOWER study findings revealed an attrition rate of 13% (n=19) [18]. In addition, some of the key measures applied in PMOMS were generated based on constructs from EMPOWER, which are to be detailed in a later section.

Furthermore, 2 pilot studies were conducted among a cohort of GDM^2^ participants as a means to demonstrate feasibility of recruitment, data collection, and technology infrastructure. The PregEMA pilot study [42] was conducted during October 2015 to January 2016 as an ancillary study to the GDM^2^ trial in a sample of pregnant women [11] to determine the feasibility of EMA/GMA data collection among pregnant women via Web-based surveys accessed via smartphones over a 4-week period. Feedback from pilot participants’ exit interviews provided valuable insight into the study elements, such as the maximum tolerable number of EMA prompts delivered in 1 day or the content of the survey questions. This pilot also demonstrated the feasibility of recruiting participants already enrolled in the parent study (GDM^2^ trial). Findings from this pilot and the lessons learned are detailed elsewhere [42].

These same participants were also approached to participate in an extension of the pilot study, which involved responding to additional EMA surveys during the first 12 weeks of the postpartum period (PostpartumEMA pilot) and reporting their weight as given on a scale provided by the study. The pilot extension further demonstrated the feasibility of participants responding to regular EMA prompts for longer periods of time and after childbirth. These surveys included key questions about participants’ physical and emotional health during the postpartum period, along with self-reported weight measurements.

The approaches employed in the pilot studies [42] provided insight about the feasibility of using EMA and mobile technology to learn about women’s pregnancies and health in real time and in their natural environments. For example, the pilot demonstrated the utility of a Web-based versus phone-based app for collecting self-reported data of daily events and experiences, coupled with the collection of location data. The pilot data indicated that women felt an average of 1 survey per day was not overburdensome and that receiving additional surveys, depending on content, would not add more of a burden. Finally, the pilot studies were the starting point for the source population, measures, infrastructure, and tools in PMOMS.

### Postpartum Mothers Mobile Study Population: Screening, Recruitment, and Follow-Up

**Figure 1:**
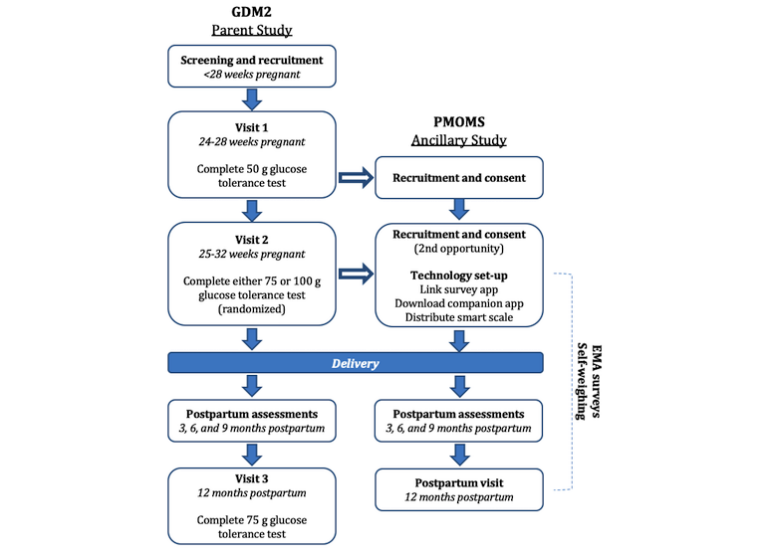
General flow of activities and data collection for the Postpartum Mothers Mobile Study (PMOMS), including the points where research activities for PMOMS and the Comparison of Two Screening Strategies for Gestational Diabetes (GDM^2^) trial intersect, as indicated by the arrows. Note that PMOMS replicates GDM^2^ protocols for the postpartum assessments and final study visit. EMA: ecological momentary assessment.

As previously described, PMOMS benefitted from the study infrastructure established by GDM^2^, given the parent study’s eligibility criteria and research aims. Figure 1 shows the key research activities and points of participant interaction from the GDM^2^ trial in relation to PMOMS. The first visit involves obtaining written informed consent to participate in GDM^2^, with consenting participants completing a nonfasting 50 g glucose tolerance test (GTT). During the hour that participants are waiting to have their blood glucose drawn, PMOMS staff approach GDM^2^ participants to potentially recruit and consent into PMOMS. All eligible GDM^2^ participants are asked to return for a second visit 1 to 2 weeks later, representing another opportunity to recruit participants into the PMOMS if they did not provide consent during the first visit.

Specific to the research activities for PMOMS, the second GDM^2^ visit is primarily used to provide the consented PMOMS participants with study materials (eg, smart scale and smartphone), additional instructions, and further information after screening is complete. During the second GDM^2^ visit, participants have about 1 to 2 hours of downtime when they are waiting to complete the 75- and 100-g oral GTTs. This visit allows ample time for setup and orientation of participants to the PMOMS devices.

A portion of women recruited into the GDM^2^ trial are not followed up after delivery because of the parent study’s sampling method for postpartum follow-up (ie, only a portion of the women with normal glucose results) [11,41]. Consequently, GDM^2^ will not conduct postpartum telephone surveys or call these participants back for a third visit (eg, 12 months postpartum). To maintain continuity and postpartum follow-up of all participants recruited into PMOMS, we implemented a study protocol similar to that of GDM^2^ for postpartum follow-up, as illustrated in Figure 1. This will ensure that all women recruited into PMOMS are followed through 12 months postpartum, regardless of whether the GDM^2^ trial follows these women after delivery. The follow-up measures include telephone-administered surveys completed at 3, 6, and 9 months postpartum, as well as the 12-month postpartum follow-up visit at the clinic.

### Smartphones, Smart Scales, and Compensation for Postpartum Mothers Mobile Study

PMOMS is designed to use smart technologies as the main tools for data collection and communication. Participants use smartphones to complete surveys on a daily basis via the PMOMS Web-based app, as well as Bluetooth-enabled smart scales for collecting weight. The process and infrastructure for these tools are described in more detail later.

PMOMS offers participants the option of using their own mobile device or to obtain a new smartphone in the event that their personal phone is not compatible with the study infrastructure or limited in its ability to complete daily surveys. We determine the compatibility of their personal phone with a basic technology screening questionnaire, which asks the participant about their smartphone usage, access to Wi-Fi at home and/or work, and whether or not they pay for an unlimited data plan. For example, if a participant expresses having an outdated mobile phone or inadequate service connection at home, our researchers recommend that they accept a new smartphone to participate in the study.

Each participant receives a smart scale, which is Bluetooth and Wi-fi enabled. It has a companion smartphone app. The Bluetooth and wireless features enable direct communication with the user’s app, logging weight and body composition data automatically and often in real time. Any weight data collected while the participant is *offline* is stored and later updated in the database when a wireless connection is available. Previous studies have validated the use of smart scales in research settings [43-46], and additional studies cite the use of other smart devices that assess anthropometric measures, such as a Bluetooth-enabled glucometer [47,48].

PMOMS compensation includes a combination of direct payments and options for receiving a new smartphone. Participants have 2 options with regard to the smartphone: (1) use their personal phone to facilitate data collection or (2) accept a new smartphone from our study as their primary device. The study finances the smartphones distributed under the second option, including an unlimited data plan, talk, and text for the duration of the study. Participants become eligible for additional monetary compensation at various points in the study, contingent on their completion of a set percentage of surveys. For participants not selected for GDM^2^ follow-up in the postpartum period, PMOMS compensates them using the same rates as the parent study. These details are reported elsewhere [11,41]. At the conclusion of PMOMS, participants are able to keep the smart scale as well as the smartphone provided by the study (if applicable).

### Daily Ecological Momentary Assessment Data Collection Protocols

**Figure 2:**
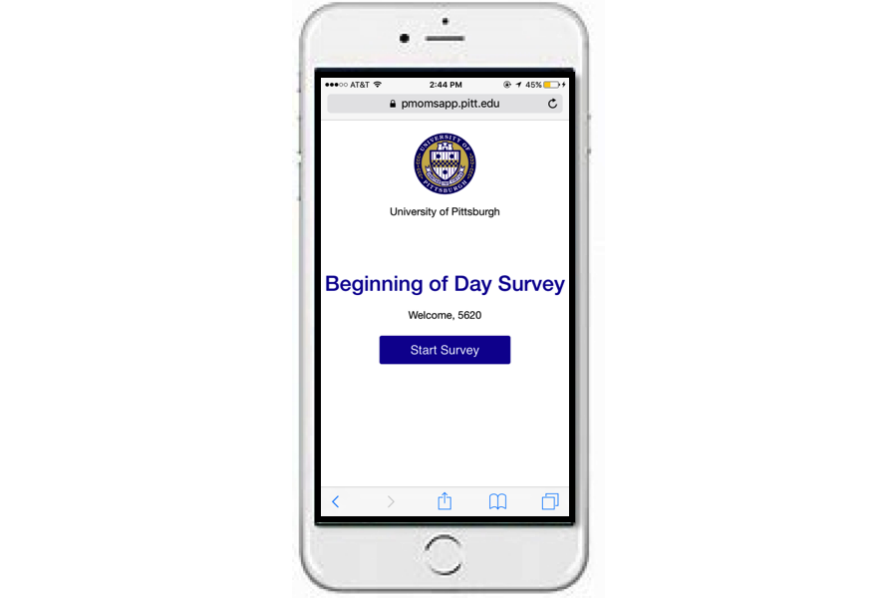
Recreated screenshot of Postpartum Mothers Mobile Study app prompting a participant to complete their beginning of day survey.

PMOMS applies 2 types of EMA data collection methods to administer surveys to participants: *signal-contingent* and *time-contingent* prompts. *Signal-contingent* responses, also known as *random*, are prompted according to a known random sampling design to obtain a representative sample of the participants’ time in the study; this is described in more detail in the next section. *Time-contingent* responses are elicited at fixed times during the day, labeled as *beginning of day* (BOD) or *end of day* (EOD) prompts. These *time-contingent* prompts are programmed according to participant preference in the PMOMS, with the only requirement being that the BOD prompt occurs at least 9 hours before the EOD prompt. Figure 2 below is an example of how the app appears to a participant on their smartphone.

PMOMS does not include *event-contingent* responses, which are initiated by the participant. In the context of the EMPOWER study (described earlier), researchers included these *event-contingent* prompts as primary outcomes typically within moments of a predefined event (eg, in EMPOWER, being tempted to eat outside of meal/diet plan); but this is not the focus of PMOMS [18]. Low participant utilization of these responses in the EMPOWER study further justified the decision not to incorporate *event-contingent* responses into PMOMS [18].

Nevertheless, PMOMS prioritized the development of a response infrastructure for participants that expressed feelings of depression or thoughts of harming themselves or others in *signal-contingent* assessments. This would not qualify as an *event-contingent* response, as it is not prompted by the participant. Instead, this inquiry into participant mood or depression occurs within a specific item in a BOD survey, which is then followed up by the study team member when appropriate. In the case of a participant expressing their need for emotional support, but not expressing the impulse to harm oneself or another, the survey app alerts study investigators such that the participant may receive proper follow-up, including the phone number for a local crisis hotline, called Resolve Crisis Network [49]. If a participant was to express any impulse to hurt themselves or others, study investigators would be alerted to contact the crisis hotline directly. In addition, any confirmation of depression or potential for self-harm would be reported to the participant's healthcare provider via the electronic medical record system.

### Sampling Design for Ecological Momentary Assessment Prompts

The delivery of survey prompts was carefully programmed according to a specific sampling design. The frequency of EMA sampling for PMOMS was informed by the time scale of temporal dependence in study variables, the relative importance of variables to study aims, and the need to reduce participant burden. Sampling too frequently, for example, will not only increase the burden on study participants but may also result in redundant data because of temporal correlation in participant responses. However, the precision (SEs) of estimated mean levels of temporally varying study outcomes, and model parameters that depend on those outcomes, decrease with decreasing sampling frequency. PMOMS researchers modeled their EMA sampling approach after the study by Shiffman [50], who partitioned variables that influence behavior into 3 categories based on time scales at which they vary from *enduring* traits, which are relatively stable, to *momentary* states, which are volatile and transient. In between the 2 extremes, there are *background conditions* “which are neither as stable as traits nor as volatile as states” [50].

**Figure 3:**
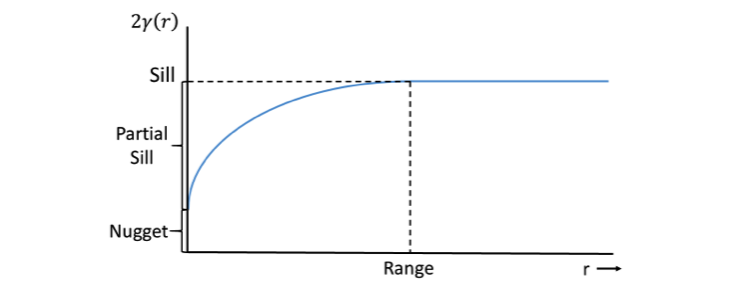
Plot of the variogram 2γ(r), an increasing function of lag time r between pairs of observations of a specified outcome.

To better understand the sampling frame applied in the EMA context more generally, and in PMOMS specifically, it is helpful to describe the underlying statistical properties. The volatility of an outcome may be described by its variance, whereas its stability may be described by the range of temporal correlation beyond which observations are uncorrelated. Borrowing from geostatistical methods [51], both volatility and temporal dependence in a time-varying outcome *Y(t)* at time *t* may be described through the variogram *2y(r)*, a function of the lag time between pairs of observations of that outcome *r* units of time apart. The variogram *2y(r)* is defined to be equal to the mean of the squared difference *[Y(t+r) – Y(t)]*^2^ between observations *Y(t)* and *Y(t+r)* that are *r* units of time apart. The variogram *2y(r)* is generally an increasing function of time lag *r* between observations (Figure 3), leveling off at an asymptote when the distance *r* attains the range of temporal correlation. Sampling at intervals closer than the range will result in redundant observations as they are temporally correlated: the higher the sampling frequency, the greater that redundancy.

The height of the plateau, or asymptote, is twice the variance, and so can be regarded as a measure of volatility. If the outcome *Y(t)* varies continuously over time, and there is no measurement error in its observations, then the variogram will approach zero as the lag distance *r* approaches zero. In many cases, however, the variogram will approach a value greater than zero, the so-called nugget effect, as lag distance *r* approaches zero. This nugget effect can be attributed to measurement error or small-scale discontinuities in the data that might arise. For example, abrupt changes in psychological states attributed to discrete events in a participant’s day, such as receipt of good or bad news, might cause this effect. For some time-varying variables, the nugget may be close to the sill, in which case, observations can be treated as approximately independent outcomes.

The variogram can be estimated using the classical variogram estimator, where the sum is the overall pairs of observations *Y(s)* and *Y(t)* approximately *r* apart in time and *N*_r_ is the number of such pairs of observations [51]:


*2*

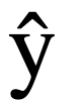

*(r)*=
^1^/
_Nr_
*∑*
*{Y(s) – Y(t)}*
^2^

**Figure 4:**
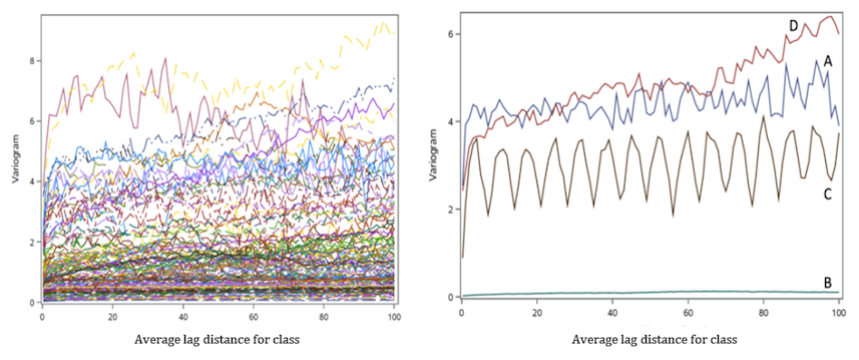
Left panel: a spaghetti plot of the variograms for confidence from random assessments of each of the Advancing Real-Time Data Collection with Adaptive Sampling and Innovative Technologies participants. Right panel: variograms for 4 select participants (A-D).

To inform the processes and EMA sampling frame for PMOMS, data from the EMPOWER study [18] were used for 2 reasons: EMPOWER served as an exemplar study in its approach to EMA data collection, as previously described; and several of the core constructs and measures from EMPOWER were applied in PMOMS. Using EMPOWER data, variograms were separately estimated for each participant in the study. On the left-hand side of Figure 4, a spaghetti plot of the variogram estimates is presented for “How confident are you that, if you have an urge to go off your healthy lifestyle plan, you can resist the urge?”, a measure of self-efficacy using a 10-point Likert scale in the EMPOWER study. Subjects show a wide variation in their time scales, especially with respect to the sills, suggesting that the volatility of self-efficacy depends on the study participant.

In addition, 4 common patterns are illustrated in the plot on the right-hand side of Figure 4. The variogram for participant A shows a short range of temporal dependence of about 3 days, suggesting that answers 3 or fewer days apart are redundant. There is a substantial nugget effect of 2.5, suggesting that there is considerable variation in self-reported confidence within days, and the sill is about 4.4, yielding an estimated variance of 2.2 for this participant. The variogram for participant B was typical of many of the participants, remaining close to zero at all lag distances. This suggests that this participant’s self-reported level of self-efficacy/confidence was nearly constant throughout the study; close examination confirms that 88.0% of the time, the participant rated confidence as *8*, and 11.7% of time, it was rated as *7*. Participant C showed a cyclic pattern, with peaks 7 days apart, suggesting that the participant’s confidence depends on the day of the week. Finally, the variogram for participant D continues to increase with increasing lag distances up to 100 days; such variograms suggest a long-term trend in the level of self-efficacy/confidence. These measures in EMPOWER related to self-efficacy/confidence are similar to measures included in PMOMS; hence, the rationale for examining temporal trends and patterns as a means to inform the sampling strategies and frequency for PMOMS EMA measures.

On the basis of previous studies and analyses of EMA data from EMPOWER, mood constructs (eg, anger, depression, and enthusiasm) have ranges of temporal correlation of less than 1 day; sleep variables have ranges of 1.6 to 2.8 days; and self-efficacy variables have ranges of 7.8 to 9.8 days [18]. These results suggest that mood shows great volatility and so should be sampled frequently, whereas self-efficacy is relatively stable and need not be sampled as frequently. On the basis of previous work [18], we targeted a mean of 1 random assessment per day in addition to the BOD and EOD surveys. The random assessment times are selected according to a self-correcting point process [52], yielding a mean of 1 assessment per day, a sampling frequency compatible with the above variogram analyses. This means that some days may deliver 0 random prompts, whereas other days can range between 1 and 3 random prompts. The self-correcting process yields a more regularly spaced pattern of random prompts and less variability in the number of random prompts per day than completely random prompts, thus reducing burden on the study participants.

To further reduce burden on the study participants, a double-sampling design [53] was implemented, under which questions regarded as being critical to study aims and important covariates (eg, racism, discrimination, self-efficacy, control, and stress) are asked during all random assessments, whereas a subset is to be asked only in randomly selected assessments (eg, mood, general wellness, and depression). Initial assessment probabilities for the latter are set to 50%.

Questions of interest can make the EOD surveys lengthy as they address the days’ physical activities, diet, and breastfeeding behaviors (postpartum only). As a result, randomized block designs were implemented as a means to query subsets of questions in each EOD survey. The prenatal period was partitioned into 28-day blocks, starting on the first Monday following enrollment, and ending at the time at which women go into labor. In each 28-day block, 4 weekend days and 10 weekdays are selected for randomized EMA items that cover the additional food-related and physical activity survey content. Half of the selected weekend days and half of the selected weekdays were randomly assigned to assessments focused on food-related questions, whereas the remainder of the selected days were physical activity assessment questions. Given the additional survey items related to breastfeeding, we use a 42-day block to cycle through the questions asked in EOD surveys to accommodate for the additional content during the postpartum period. A total of 4 weekend days and 10 weekdays are selected in this block to cover both food-related and physical activity questions. Similarly, 4 additional weekend days and 10 weekdays are selected to cover food-related and breastfeeding questions. Finally, the remaining 4 weekend days and 10 weekdays are assigned physical activity and breastfeeding questions.

The participants have a break from answering any prompts from delivery through 7 days postpartum, to allow them time to become acclimated to their new family circumstances. Postpartum assessments continue after that 7-day period

### Ecological Momentary Assessment Survey Questions

We assess numerous constructs and measures in the BOD, EOD, and random prompts, as illustrated in Table 1. These measures were selected based on their hypothesized associations with postpartum weight retention and, specifically, contributors to the racial disparities. In many cases, these previously validated scales (eg, Gendered Racial Micro aggressions Scale) have not been applied in an EMA context; therefore, PMOMS is attempting to apply such measures and constructs in ways that previous studies have not. By assessing the participants’ experiences through these EMA measures longitudinally, PMOMS expects to gain a more nuanced understanding of how contextual, behavioral, and psychosocial factors intersect to explain changes in postpartum weight and cardiometabolic health and, more specifically, how they contribute to existing racial disparities.

Racism and discrimination are measured in multiple ways, including the specific construct identified in Table 1. The participants also answer a series of 12 items related to microaggressions, based on the Gendered Racial Microaggressions Scale [28,29], experienced that day such as, “Receive negative comments about my skin tone,” “Someone made me feel exotic because of my race or gender,” and “Someone made a sexually inappropriate comment towards me.” If they answer yes to any of the items, follow-up questions will inquire about their feelings, their reactions, and the location of the interaction.

In addition to self-weighing (described further below), participants are asked questions about context such as “Where are you located?” and “Who are you with?” Each prompt also asks for permission to capture GPS location (geospatial data and approaches are also described further). These 3 context-related measures are asked in all survey prompts, including BOD, EOD, and random.

**Table 1:** Primary variables and covariates assessed in ecological momentary assessment prompts, with examples.

Construct	Delivery	Measurement example
Sleep [54]	BOD^a^	How long (in minutes) did it take you to fall asleep last night?
Diet [55]	BOD, EOD^b^	How many meals did you eat today?
Sedentary/physical activity [56]	EOD	How many hours did you spend sitting today?
Racism [31]	Random	How often were you treated with less courtesy than other people because of your race? (0=never; 1=almost never; 2=sometimes; 3=fairly often; 4=almost every day).
Stress [57]	Random	How often have you felt nervous or stressed? (0=never; 1=almost never; 2=sometimes; 3=fairly often; 4=very often).
Control [57]	Random	How often have you felt you were able to control important things in your life? (0=never; 1=almost never; 2=sometimes; 3=fairly often; 4=very often).
Self-efficacy [57]	Random	How often have you felt confident about your ability to handle your personal problems? (0=never; 1=almost never; 2=sometimes; 3=fairly often; 4=very often).
Depression [58]	Random	Have you felt depressed today? (yes or no)
Mood	Random	How are you feeling? (eg, content, tired, hungry).
Support	Random	Please rate the level of support you have to care for yourself. (0-4).
Breastfeeding	Random	Did you breastfeed today? (yes or no).

^a^BOD: beginning of day.

^b^EOD: end of day.

### Non-Ecological Momentary Assessment Survey Questions

In addition to the many EMA-based surveys, PMOMS also incorporates surveys into the app to conduct assessments that are not temporally or ecologically based. These *non-EMA* prompts are delivered subsequent to BOD surveys at specific milestones throughout the study. Table 2 describes these non-EMA surveys.

In addition to data collection via smartphones and scales, key measures and constructs are collected via the larger GDM^2^ trial. This includes a series of questionnaires during the GDM^2^ screening process, baseline (during visit 1), randomization visit (visit 2), delivery visit, and at 12 months postpartum (visit 3). As described in the section *PMOMS Recruitment, Retention, and Follow-up,* PMOMS researchers replicate the same postpartum protocol for any participant not selected for follow-up by GDM^2^. The survey measures collected during the GDM^2^ study visits and telephone calls address stress, mood, depression, physical activity, diet (eg, 24-hour dietary recall), and demographic information. Additional measures of pregnancy and infant health outcomes are abstracted from the electronic medical records.

**Table 2:** Timing and content of non-ecological momentary assessment surveys delivered after beginning of day prompts throughout the study.

Delivery period	Constructs measured
14 days after study enrollment	Technical issues with devices or app Burden of EMA^a^ prompts Experiences of discrimination over lifetime [31]
Day 8 after delivery	Anxiety in interpersonal relationships Social support Use of social media to connect with peers Breastfeeding initiation
Every 3, 6, and 9 months after delivery	Burden of EMA prompts Issues not being addressed in surveys Participant behaviors related to weight loss
After final study visit (1 year postpartum)	Participant history of residence(s) Workplace/employment Social support Satisfaction with Postpartum Mothers Mobile Study

^a^EMA: ecological momentary assessment.

### Self-Monitoring With Smart Scales and Health Mate App

Although the smart scale and app provide a convenient platform, researchers have little control over how participants choose to use the devices’ additional features. One instance of this occurred during study development, when the smart scale manufacturers added a *pregnancy mode* to their app, including additional counseling and reminders that correspond to the user’s gestational age. If the pregnancy mode is activated, the app will present dietary recommendations or advice on gestational weight gain that could influence a participant’s behavior and could subsequently bias the study outcomes. Consequently, although we do not have direct control over access to this information, we provide instructions upon enrollment for the participants to avoid using this feature because of limited information about the sources and validity of the health and behavioral information provided to them.

Via the PMOMS app, we prompt all the participants, starting each Friday and throughout the weekend, to remind them to weigh themselves with the question, “Were you able to weigh yourself and get a weight?” If they answer no, then we ask them to “Please describe why.” Response options include, “Scale is not working properly” and “I did not want to weigh myself.” Early on in the study development, we identified the potential influence of monitoring one’s weight on the participant’s behavior during the study and, thus, their outcome measurements. The participants are only prompted to step on the scale weekly; however, the participants may choose to weigh themselves more often, as the scale is available to them in their homes. The Self-Monitoring and Recording Using Technology trial investigators [59] described self-monitoring as the *cornerstone* of behavioral treatment. Consequently, participant behaviors related to self-weighing and perinatal weight management are taken into account when evaluating factors that are hypothesized to affect weight and cardiometabolic measurements, as described in the previous section regarding non-EMA surveys.

### Postpartum Mothers Mobile Study Technology and Infrastructure

The PMOMS Web-based app has 3 core components: data collection, data management, and data analytics. Each component is designed to maximize the study aims, particularly the unique features of EMA data collection throughout pregnancy, as well as leverage the Bluetooth technology in the smart scale for repeated weight measures. This section provides additional detail on the technical infrastructure established by PMOMS to ensure efficient and secure data collection.

The technology infrastructure has 5 major modules: (1) an administration module to invite participants to the study, manage and modify participants’ profiles, and authenticate PMOMS for using the scale; (2) a Web-based survey module that contains specific questions related to participants’ circumstances at different locations and times; (3) a database management module for storing and managing the collected data as well as generating parameters for the survey using the scheduled tasks; (4) a random value generator module to provide constrained random times for random assessments and EOD block group questions; and (5) a data retriever module to fetch body measurement data from the third-party scale database.

#### App Architecture and Data Flow

**Figure 5:**
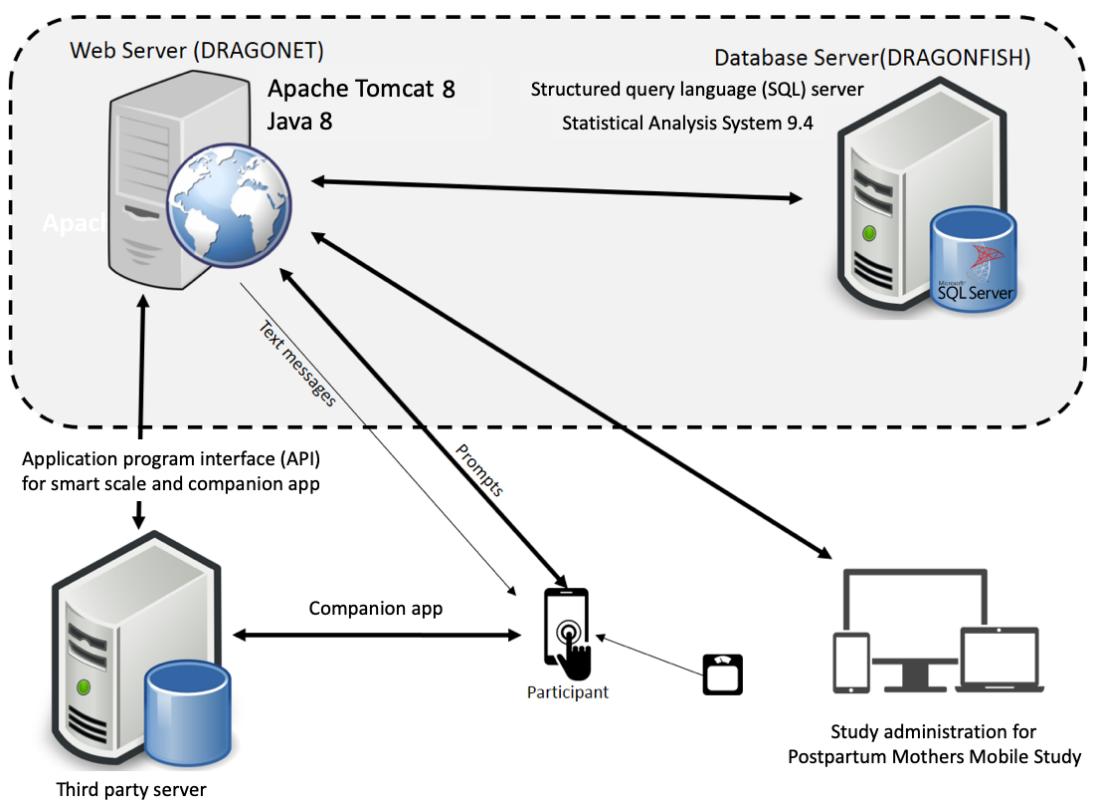
The 4-tier architecture of Postpartum Mothers Mobile Study application and technology infrastructure.

PMOMS uses a mobile app based on a client-server architecture with 4 tiers: presentation, logic, database, and scale (Figure 5).

The presentation tier consists of interfaces used to communicate the surveys, management panel, data, and responses of the system to end users of the app, including participants and staff. The content that is communicated with the end user consists of both static and dynamic information; an example of the latter includes survey questions based on stage of participants and time. The administrative interfaces are designed for desktops and tablets, allowing administration staff to manage participants’ information.

Communication between the users’ phones and the server is secured through the transport layer security protocol, previously known as the secure sockets layer protocol, whereas communication between the servers is secured using the firewall system implemented by the Computing Services and Systems Development office within the University of Pittsburgh. These interfaces for participants (ie, smartphones) and staff (ie, tablet and desktop) are shown in Figure 5.

The logic tier includes the essential logic to ensure that all functions in PMOMS are performed consistently and according to the design specifications. Furthermore, rules and algorithms for evaluating participants’ circumstances and for compiling the questionnaires are all conducted in this tier. See the logic tier (ie, Dragonet) in Figure 5. Surveys are received by participants at scheduled times, so the data go from the logic tier to presentation tier (smartphone). For the purpose of loading scheduled surveys on the participants’ smartphones, a text message containing a URL linking the surveys is sent to the stored contact information for participants. Reminders about the survey are sent to respondents if they do not complete the surveys within a specified amount of time. In the case of time-contingent prompts (eg, BOD and EOD surveys), participants have 30 min to complete the survey once it is delivered; signal-contingent prompts (eg, random) allow participants 60 min for completion. The URL expires after these time periods, which triggers a text message indicating cancellation of the survey to be sent to the participant. If a participant does not start the survey while it is valid, a record of *not attempted* is automatically entered into the database and the survey is no longer available. However, a survey that was started or ongoing, but not submitted in the time window, is still valid for submission for an additional hour. Survey responses received in the logic tier are then processed for storage in the database tier.

The database tier contains a database management system for storing and managing all data collected in PMOMS. Data collected and managed in this tier are participants’ information and responses to the surveys. See the database tier (ie, Dragonfish) in Figure 5.

Data received from the smart scale by the presentation tier, through Wi-Fi or Bluetooth connection with the scale, is then sent to the scale tier, which consists of a third-party database, maintained by the producers of the smart scale and accompanying app, as a repository of body measurements of PMOMS participants from the smart scale. As the PMOMS team is authorized by participants to access their data, token keys are generated and stored in the database. The required and authorized data are transferred from this tier to the database tier on a regular basis using the OAuth framework, which is an open protocol allowing for limited but secure communication between multiple applications [60]. Finally, the database tier retrieves the weight data from the scale tier. See the scale tier in Figure 5.

#### Geolocation

**Figure 6:**
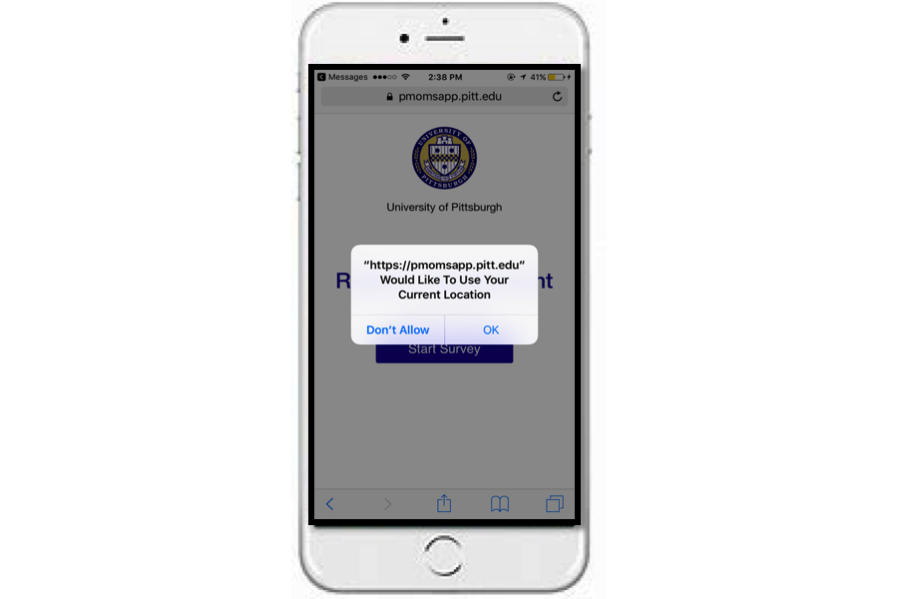
Recreated screenshot of Postpartum Mothers Mobile Study survey app requesting permission to collect geolocation data.

As described previously, the Web-based, platform-independent app includes GPS capabilities, requesting participant permission to record GPS coordinates whenever any survey prompt is received. Figure 6 illustrates how the app requests permission. Once granted, the participant’s device location is provided to the app through the HTML5 Geolocation application programming interface. To protect the participant’s privacy, location data are transmitted through a secure connection.

Although other studies have attempted to match GMA data with timestamps for EMA responses [39,61], PMOMS staff developed a Web app that delivers EMA prompts and collects GPS coordinates simultaneously.

#### Data Management

The database tier of PMOMS is responsible for storing and managing data, which are mostly the responses to the various survey questions that are either required or optional. All participants must answer the required questions, and the optional questions can be skipped and are recorded as missing values in the database.

For efficient data management, the missing values are coded in different ways indicating the reason for their absence. Example codes are *not applicable*, *missed*, *not asked*, and *unknown*. These missing data can be used for generating reports to track the level of participation and monitor the integrity of the database. In addition to the storage of data in the database, a copy of the data is stored in a flat file for cross-validation of the responses and as a backup in case of server crashes.

### Overview of Analytical Strategy: Understanding Postpartum Weight Change

PMOMS aims to predict postpartum weight retention in part from mean levels of time-varying variables *x(t)* attained using EMA according to the formula:


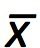
*(T)=*^1^/_T_*(∫*_0_^T^)*x(t)dt

Here, the integral is over the sets of time either during pregnancy or postpartum (or the entire study period), and 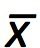
*(T)* may be regarded as a population mean where the population includes all points in time in the interval *[0,T]*.

Random assessment provides a representative sample of times from which design-unbiased estimates [62] of 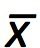
*(T)* may be obtained, where *Π(t)* is the sampling intensity at time *t*, and the sum is overall random assessments *S*_T_ in the interval [63]. Missing data may be addressed using the weighted estimator, where *1 – q(t)* is the probability that an observation at time *t* is missing.

If data are missing completely at random, then *q(t)* is constant and may be estimated empirically by the proportion of data that are not missing. Otherwise, *q(t)* may be estimated (eg, using a regression model) as a function of the observed data. Then the mean level of the time-varying variable may be estimated using:


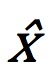
*(T)*=^1^/_T_*∑*_t∈ST_*x(t)/Π(t)q(t)

The EMA sampling intensity, targeting a mean of 1 random assessment per day (as described previously), was used to balance the precision of estimating mean values of EMA predictors against the burden of study participants. With respect to the latter, burdensome EMA assessments may not only adversely impact compliance with EMA assessments and quality of responses to those assessments but also act as an intervention impacting participant behavior. As it estimates 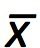
*(T)* with error, replacing with 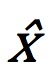
*(T)* can result in biased estimates of regression coefficients in both linear and nonlinear models [64,65]. This measurement error is negligible if the variance of 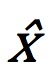
*(T)* is small within subjects compared with the variance of 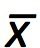
*(T)* between subjects.

Secondary data analyses will include prediction of EMA outcomes using linear mixed-effects and generalized linear mixed models, including random subject effects. Such models typically assume that the within-subjects variance component does not depend on subject. The variogram analyses, including the analyses described earlier of the EMPOWER data, suggest that there is considerable variation in the within-subjects variance among subjects. Therefore, we plan to construct mixed-effects models in which the within-subjects variance depends on the participant.

## Results

By November 2017, the PMOMS app was completed based on the prototype from the previous pilot studies and extensive testing with volunteers and study staff. PMOMS recruitment and data collection began December 2017 with an expectation to continue recruitment through September 2019 and conclude data collection in September 2020. Initial results are expected December 2020. As of early May 2019, PMOMS screened and approached 356 participants and 305 consented to participate. Out of those, 284 have been issued devices (smartphones and/or smart scales) and have been entered into the PMOMS technology systems to begin completing EMA surveys and collecting weight data. So far, 266 participants have given birth and are engaged in postpartum assessments and follow-up. On the basis of baseline data generated from GDM^2^ in April 2019 that were available for 238 participants, 63% of the population was white; 25% was black; 4% was Asian; 3% identified as multiracial; and the remaining identified as Native Hawaiian, American Indian, or another racial category (not specified). Out of the 238 participants, 7.2% identified as Hispanic/Latino.

As of early May 2019, the attrition rate was approximately 15% because of withdrawal from GDM^2^ or PMOMS for various reasons including moving to new locations, health challenges, or lack of interest in continued participation. PMOMS continuously monitors study recruitment and attrition and has instituted procedures to continue engaging participants, such as check-in calls to discuss any technology challenges, breaks from continuous EMA prompts as needed, and a *Contact Us* button within the app for reporting technological challenges. In addition, a scale back of EMA surveys will be implemented to further reduce participant burden. On the basis of data generated in May 2019 (approximately 17 months after study recruitment began), survey completion rates during the first 4 weeks of study participation were 77.7% overall (76.4% for BOD, 78.6% for EOD, and 78.2% for random). The overall survey completion rates during the entire pregnancy period were 76.6% (74.3% for BOD, 77.8% for EOD, and 77.7% for random) and declined slightly to 69.5% at 1 month postpartum, 66.2% at 2 months postpartum, 64.2% at 3 months postpartum, 61.8% at 4 months postpartum, 62% at 5 months postpartum. We do not report completion rates at 6 months and higher because of smaller sample sizes at that time period.

## Discussion

The PMOMS includes multiple processes for study initiation, development, and implementation. This study is unique in that it attempts to engage populations during late pregnancy and through 1 year postpartum to enhance our understanding of racial disparities in postpartum weight and cardiometabolic health by leveraging an existing trial aimed to understand how GDM^2^ testing strategies and outcomes may influence gestational diabetes, metabolic issues in after pregnancy, and infant outcomes. The work generated from PMOMS coupled with GDM^2^ can also provide insight into health outcomes over the life course irrespective of pregnancy status.

A key strength of this study is the implementation of novel measurements of stress, discrimination, behavior, and context. We apply an intensive EMA protocol that calls for daily participation over an average of 15 months per woman. To our knowledge, this is the first study to specifically engage women over this length of time, using an EMA app via mobile technology. Through a period of development and internal testing, we were able to maximize data collection and minimize error. In addition, the use of the smart scale to capture weight allows for measurements of weight over time and during time periods that have not been captured by previous studies. Finally, it serves to highlight the PMOMS protocol’s leveraging of recruitment infrastructure in GDM^2^, which facilitates the enrollment of participants by consolidating staff support from the 2 studies, resulting in a less costly and more efficient protocol.

The PMOMS design and app have utility not only among pregnant populations and related to weight and cardiometabolic health but also among other populations and health conditions, particularly in understanding phenomenon that may change frequently or that change over time. The knowledge gained can help identify factors that influence obesity and cardiovascular disease disparities for women, inform the refinement of existing interventions, and provide insights for the development of novel approaches that incorporate evolving technology that permits timely, bidirectional communication between women, support systems, and providers throughout pregnancy and during the postpartum period.

## Abbreviations

BOD: beginning of day
EMA: ecological momentary assessment
EMPOWER: Advancing Real-time Data Collection with Adaptive Sampling and Innovative Technologies
EOD: end of day
GDM ^2^: Comparison of Two Screening Strategies for Gestational Diabetes
GMA: geographic momentary assessment
GPS: geographic positioning system
GTT: glucose tolerance test
PMOMS: Postpartum Mobile Mothers Study
